# Accessing the syndemic of COVID-19 and malaria intervention in Africa

**DOI:** 10.1186/s40249-020-00788-y

**Published:** 2021-01-07

**Authors:** Benyun Shi, Jinxin Zheng, Shang Xia, Shan Lin, Xinyi Wang, Yang Liu, Xiao-Nong Zhou, Jiming Liu

**Affiliations:** 1 School of Computer Science and Technology, Nanjing Tech University, Nanjing, 211800 Jiangsu China; 2 National Institute of Parasitic Diseases, Chinese Center for Disease Control and Prevention, Shanghai, 200025 China; 3 Key Laboratory of Parasite and Vector Biology, Ministry of Health, Shanghai, 200025 China; 4 Chinese Center for Tropical Diseases Research, Shanghai, 200025 China; 5 WHO Collaborating Centre for Tropical Diseases, Shanghai, 200025 China; 6 National Center for International Research on Tropical Diseases, Ministry of Science and Technology, Shanghai, 200025 China; 7 School of Global Health, Chinese Center for Tropical Diseases Research, Shanghai Jiao Tong University School of Medicine, Shanghai, 200025 China; 8 College of Information Engineering, Nanjing University of Finance & Economics, Nanjing, 210003 Jiangsu China; 9 Department of Computer Science, Hong Kong Baptist University, Hong Kong, China

**Keywords:** COVID-19 pandemic, Non-pharmaceutical interventions, Particle Markov chain Monte Carlo, Insecticide-treated nets, Vectorial capacity, Malaria transmission potential

## Abstract

**Background:**

The pandemic of the coronavirus disease 2019 (COVID-19) has caused substantial disruptions to health services in the low and middle-income countries with a high burden of other diseases, such as malaria in sub-Saharan Africa. The aim of this study is to assess the impact of COVID-19 pandemic on malaria transmission potential in malaria-endemic countries in Africa.

**Methods:**

We present a data-driven method to quantify the extent to which the COVID-19 pandemic, as well as various non-pharmaceutical interventions (NPIs), could lead to the change of malaria transmission potential in 2020. First, we adopt a particle Markov Chain Monte Carlo method to estimate epidemiological parameters in each country by fitting the time series of the cumulative number of reported COVID-19 cases. Then, we simulate the epidemic dynamics of COVID-19 under two groups of NPIs: (1) contact restriction and social distancing, and (2) early identification and isolation of cases. Based on the simulated epidemic curves, we quantify the impact of COVID-19 epidemic and NPIs on the distribution of insecticide-treated nets (ITNs). Finally, by treating the total number of ITNs available in each country in 2020, we evaluate the negative effects of COVID-19 pandemic on malaria transmission potential based on the notion of vectorial capacity.

**Results:**

We conduct case studies in four malaria-endemic countries, Ethiopia, Nigeria, Tanzania, and Zambia, in Africa. The epidemiological parameters (i.e., the basic reproduction number $R_0$ and the duration of infection $D_I$) of COVID-19 in each country are estimated as follows: Ethiopia ($R_0=1.57$, $D_I=5.32$), Nigeria ($R_0=2.18$, $D_I=6.58$), Tanzania ($R_0=2.47$, $D_I=6.01$), and Zambia ($R_0=2.12$, $D_I=6.96$). Based on the estimated epidemiological parameters, the epidemic curves simulated under various NPIs indicated that the earlier the interventions are implemented, the better the epidemic is controlled. Moreover, the effect of combined NPIs is better than contact restriction and social distancing only. By treating the total number of ITNs available in each country in 2020 as a baseline, our results show that even with stringent NPIs, malaria transmission potential will remain higher than expected in the second half of 2020.

**Conclusions:**

By quantifying the impact of various NPI response to the COVID-19 pandemic on malaria transmission potential, this study provides a way to jointly address the syndemic between COVID-19 and malaria in malaria-endemic countries in Africa. The results suggest that the early intervention of COVID-19 can effectively reduce the scale of the epidemic and mitigate its impact on malaria transmission potential.

## Background

In 2020, the coronavirus disease 2019 (COVID-19) caused by severe acute respiratory syndrome coronavirus 2 (SARS-CoV-2) spread across the world and resulted in a pandemic [1–3]. Even though great efforts have been made, the pandemic has continued and worsened in some countries. As of June 29, 2020, it has caused more than 10 million confirmed cases and 499 913 deaths in as many as 213 countries and territories [4]. To contain the global spread of COVID-19, a set of non-pharmaceutical interventions (NPIs) have been suggested and implemented, such as isolation of ill persons, quarantine of exposed persons, contact tracing, travel restrictions, school and workplace closures, and cancellation of mass gatherings [5, 6]. It is estimated that more than 138 countries have closed schools nationwide, and several other countries have implemented regional or local closures [7]. An integrative literature review has shown that the COVID-19 pandemic may have a great socio-economic impact on global poverty [8]. The entire world is facing a human, economic and social crisis [9–11]. According to the World Health Organization, there is an urgent need to aggressively tackle the COVID-19 pandemic while ensuring that other diseases, such as malaria, are not neglected.

Malaria is a mosquito-borne infectious disease, which has long been a nightmare for countries in sub-Saharan Africa. Based on the WHO’s world malaria report 2019, sub-Saharan Africa accounted for about 93% of all malaria cases and 94% of deaths in 2018, from an estimated 228 million cases and 405 000 deaths worldwide [12]. As of March 12, 2020, several malaria-endemic regions in Africa have reported a few imported COVID-19 cases, which has led to serious public health emergencies. As the COVID-19 pandemic spreads in Africa, it becomes a challenging task to maintain core malaria control services while protecting health workers against COVID-19 transmission [13]. As a consequence, malaria control services have been disrupted in many malaria-endemic regions in sub-Saharan Africa, among which insecticide-treated net (ITN) campaigns have been considered as the most important measure for malaria intervention and control across Africa in the last two decades [14]. However, as of March 2020, there have been reports of the suspension of insecticide-treated net campaigns in several African countries due to concerns around exposure to COVID-19.^1^ It is estimated that under the worst situation, where all ITN campaigns were suspended, malaria deaths in sub-Saharan Africa in 2020 would reach an estimated 769 000 [15].

In this study, we aim to assess the syndemic of COVID-19 and malaria intervention in four malaria-endemic countries in Africa: Ethiopia, Nigeria, Tanzania, and Zambia. First, we adopt a particle Markov Chain Monte Carlo (PMCMC) method to estimate the epidemiological parameters in each country [16]. Based on the estimated epidemiological parameters, we can then simulate the epidemic curves of the COVID-19 in each country under various non-pharmaceutical interventions (NPIs) in terms of when and how different NPIs are implemented. Existing studies have shown that NPIs play essential roles in fighting the COVID-19 epidemic [5]. For example, Lai et al. have studied the effects of three major groups of NPIs on containing the spread of COVID-19 across China [6], including (1) contact restrictions and social distancing, (2) early identification and isolation of potential cases, and (3) inter-city travel restriction. Because in this study we focus mainly on the spread of COVID-19 in Africa at the country level, we only conduct scenario analysis on the first two groups of NPIs in the four countries. Moreover, to quantify the impact of COVID-19, we assume that the distribution of ITNs is affected by the severity of the COVID-19 epidemic. The severer the epidemic, the greater the impact on the distribution of ITNs.

There is no doubt that the cessation or disruption of ITN distribution will result in an increase in the human biting rate, and further the transmission potential of malaria. Specifically, in this study, we adopt the notion of vectorial capacity (VCAP) to characterize the transmission potential of a mosquito population in the absence of *Plasmodium*, which is defined as the number of potentially infective contacts a person makes, through the mosquito population, per day [17, 18]. Since 2012, Ceccato et al. have proposed a VCAP product to monitor malaria transmission potential in epidemic regions in Africa, where the value of VCAP is calculated by the dynamically changing meteorological factors, i.e., temperature and rainfall [19]. Based on the historical VCAP data, we can analyze the seasonal patterns of malaria transmission potential in each country. By treating the number of ITNs distributed in 2019 as a benchmark, we can then access and predict the impact of cessation or disruption of ITN distribution on malaria transmission potential under different NPI scenarios in each country. In this study, we aim to quantify the extent to which the COVID-19 pandemic in Ethiopia, Nigeria, Tanzania, and Zambia in Africa with high malaria burden could lead to the change of malaria transmission potential in 2020.

## Methods

### Country selection and data sources

**Fig. 1:**
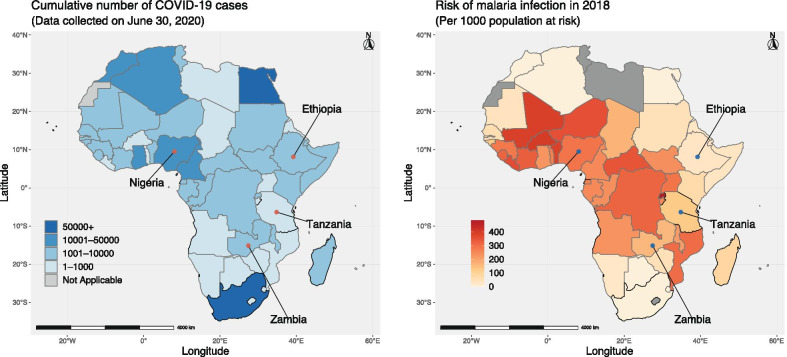
An illustration of the cumulative number of reported COVID-19 cases and the incidence of malaria infection in Africa. The left shows the total number of reported COVID-19 cases till June 30, 2020. The right shows the incidence of malaria infection (per 1000 population at risk) in 2018. The figure was generated using the Free Software R with version 3.6.3

Figure 1 illustrates the cumulative number of reported COVID-19 cases and the incidence of malaria infection in Africa. The four countries are located in eastern Africa, western Africa, and central and southern Africa, where the infection risk of both COVID-19 and malaria is relatively high. First, to estimate the epidemiological parameters of COVID-19 in each country, we use the time series of cumulative COVID-19 cases in each country till June 2, 2020. The reported COVID-19 cases in each country are collected from the website of the World Health Organization (Source: https://covid19.who.int/). Then, we characterize the annual pattern of malaria transmission potential in each country based on the historical values of VCAP from January 1, 2004, to December 31, 2019. The values of VCAP are downloaded from the International Research Institute for Climate and Society (Source: http://iridl.ldeo.columbia.edu/maproom/Health/Regional/Africa/Malaria/VCAP/index.html). Finally, to quantify the impact of COVID-19 pandemic on malaria transmission potential, we have also collected the population size, the ITN coverage, and the total number of ITNs available for each country. Based on the Malaria Indicator Survey (MIS) in each country, the ITN coverage rate before 2020 is estimated by the percentage of de facto household population who slept under an ITN the night before the survey. While the total number of ITNs available in 2020 can be obtained from the Malaria Operational Plan (FY 2019) of each country. All data and their sources are summarized in Table 1.

**Table 1:** The data used for assessing the impact of COVID-19 pandemic in Ethiopia, Nigeria, Tanzania, and Zambia

Country	Population^a^	COVID-19^b^	Malaria^c^	ITN coverage^d^	ITNs available in 2020^e^
Ethiopia	109 224 414	5846	31.814	39.70%	4 723 087
Nigeria	195 874 683	25 133	291.194	54.70%	11 300 000
Tanzania	56 313 438	509	124.265	52.20%	7 559 527
Zambia	17 351 708	1568	156.701	63.90%	1 988 000

^a^Population size: https://www.worldometers.info/population/countries-in-africa-by-population/

^b^The reported COVID-19 cases on June 30, 2020: https://covid19.who.int/

^c^Incidence of malaria (per 1000 population at risk): https://data.worldbank.org/indicator/SH.MLR.INCD.P3

^d^Malaria Indicator Survey: https://www.malariasurveys.org

^e^Malaria Operational Plan (FY 2019): https://www.pmi.gov/resource-library/mops/fy-2019

### Estimating epidemiological parameters of COVID-19 pandemic

To accurately predict the trend of COVID-19 pandemic, we first estimate the epidemiological parameters of COVID-19 by fitting the time series of reported COVID-19 cases in each country. Here, the time series is about the reported date of the COVID-19 infections as of June 2, 2020. Evidence has shown that asymptomatic infections play essential roles in the spread of COVID-19. However, due to limited public health resources in the four countries in Africa, it is difficult to identify asymptomatic infections in the early stages of the epidemic. In this case, following existing studies [26, 27], we resort to the classical susceptible–exposed–infectious–removed (SEIR) model to simulate the transmission dynamics of COVID-19 in a population of size *N*:

1 $$\begin{aligned} {\left\{ \begin{array}{ll} \frac{dS(t)}{dt} = - \frac{R_0}{D_I} \cdot \frac{ S(t) I(t)}{N}\\ \frac{dE(t)}{dt} = \frac{R_0}{D_I} \cdot \frac{ S(t) I(t)}{N} - \frac{E(t)}{D_E} \\ \frac{dI(t)}{dt} = \frac{E(t)}{D_E} - \frac{I(t)}{D_I} \\ \frac{dR(t)}{dt} = \frac{I(t)}{D_I} \end{array}\right. } \end{aligned}$$

where *S*(*t*), *E*(*t*), *I*(*t*), and *R*(*t*) represent the number of susceptible, exposed, infectious, and removed individuals at time *t*. In this study, we assume that the reported COVID-19 cases are removed or quarantined from the population and can no longer infect others. Along this line, the time series of reported cases we observed is actually the states of *R*(*t*) over time.

There are three epidemiological parameters in the SEIR model: $R_0$ is the basic reproduction number; $D_E$ is the average latent period; and $D_I$ is the average contagious period (i.e., the average duration that an infectious individual is confirmed to be infected). Following the study in [26], we assume that the latent period is the same as the incubation period. Further, based on the estimation in [1], the mean incubation period of COVID-19 was 5.2 days. In this case, we set $D_E=5.2$. The infectious rate, $\beta =R_0/D_I$, controls the rate of spread that represents the probability of transmitting disease between a susceptible and an infectious individual. In this study, we adopt the particle Markov Chain Monte Carlo method to estimate epidemiological parameters $R_0$ and $D_I$ in each country by fitting the time series of the cumulative number of reported COVID-19 cases [16, 20].

We implement the PMCMC method and simulate the COVID-19 epidemic using python programming language version 3.8.5 (source: https://www.python.org/). We assume that the first exposed case [i.e., *E*(0)] appears *d* days before the date of the first reported case in each country. According to the Bayesian inference method, uninformative uniform priors are assigned to model parameters to reduce their influence on the posteriors, that is, $R_0 \sim U(0,8)$, $D_I \sim U(1,20)$, and $d \sim U(0,30)$. Since the interval of each prior covers almost all possible values of the corresponding parameter, such settings have little effect on the inference results as long as the number of iterations is enough. With careful pre-testing, we set the proposal distribution of each parameter to be normal distribution: $q(R_0^{*}|R_0)=norm(R_0^{*}|R_0,0.5)$, $q(D_I^{*}|D_I)=norm(D_I^{*}|D_I,0.5)$, and $q(d^{*}|d)=norm(d^{*}|d,0.5)$. After initializing the values of model parameters as $R_0=2.5$, $D_I=5$, and $d=14$, we run the PMCMC algorithm with 200 particles for 100,000 iterations. Finally, the posterior of each parameter is built upon the last $80\%$ iterations with a discarded burn-in of 20,000 iterations.

### Simulating epidemic dynamics of COVID under different NPIs

Based on the estimated model parameters, we simulate the dynamics of COVID-19 under two groups of NPIs: (1) contact restriction and social distancing (e.g., contact restrictions and personal preventive actions), and (2) early identification and isolation of cases. Generally speaking, contact restrictions can reduce the infectious rate $\beta$ of COVID-19; while early identification and isolation of potential cases can reduce the average duration of infection $D_I$. On the one hand, we assess the effects of social distancing interventions by reducing the estimated infectious rate $\beta$ to 25% and 10%, while keeping the duration of infection unchanged. This is equivalent to reducing $R_0$ to 25% and 10% of its original value. On the other hand, we also evaluate the impact of combined intervention strategies, where both social distancing and early identification and isolation are implemented. Specifically, the epidemic dynamics of COVID-19 are simulated when reducing $R_0$ to 25% and $D_I$ to 2 (or 4) at the same time. In addition to what types of NPIs are implemented, it is also important when to implement the interventions. Accordingly, we further simulate the epidemic dynamics of COVID-19 under various settings of NPIs that are implemented on May 18 and June 17, 2020, respectively.

### Analyzing malaria transmission potential from vectorial capacity

We adopt the notion of vectorial capacity to evaluate malaria transmission potential in malaria-endemic countries in Africa. Based on the Macdonald model [21], the VCAP can be formulated as:

2 $$\begin{aligned} V=\frac{ma^2e^{-gn}}{g} = \frac{-ma^2p^n}{\ln p}, \end{aligned}$$

where *m* is the average mosquito density per person; *a* is the expected number of bites on humans per mosquito, per day (i.e., human feeding rate); *g* is the per-capita daily death rate of a mosquito (i.e., the force of mortality); *n* is the sporogonic cycle length of the *Plasmodium*; $p=e^{-g}$ represents the probability of a mosquito survives through one whole day. Conceptually, the VCAP incorporates all information about mosquito population (e.g., human biting rate, life expectancy), which is defined as the number of potentially infective contacts a person makes, through the mosquito population, per day. Many studies have shown that the value of VCAP can be calculated based on meteorological factors, such as temperature and precipitation [22, 23]. For example, Ceccato et al. have calculated the average vectorial capacity per 8 days for areas where malaria is considered to be an epidemic in Africa [19]. If there is no abnormal climate change, the annual pattern of malaria transmission potential in each country should be relatively stable across different years. On this basis, we download and extract the 8-day average vectorial capacity for each county from January 1, 2004, to December 31, 2019. We then use the means of the 16-year VCAP as a baseline of the annual pattern of malaria transmission potential.

### Accessing the impact of COVID-19 response on malaria transmission potential

In this study, we focus mainly on assessing the impact of COVID-19 response on the disruption of ITN distribution, and further on the transmission potential of malaria. Launched in 2005, the President’s Malaria Initiative (PMI) strives to reduce the burden of malaria across 15 high-burden countries in sub-Saharan Africa through a rapid scale-up of four proven and highly effective malaria prevention and treatment measures, including insecticide-treated mosquito nets. In most countries, the PMI has supported ITN distribution through universal mass campaigns and continuous distribution channels. Based on the Malaria Operational Plan (FY 2019) in each country, we can obtain the total ITNs available from different partner contributions in 2020 (see Table 1). In this study, we assume that the available ITNs are distributed throughout the year in a way that the number of distributed ITNs is proportional to the annual pattern of malaria transmission potential in each time interval (eight days in this study). In doing so, the newly increased number of ITNs in a specific time interval *t* in 2020 can be estimated as:

3 $$\begin{aligned} \Delta _i(t) = \frac{V_i(t)}{\sum _t V_i(t)}\Delta _i, \end{aligned}$$

where $\Delta _i$ represents the total number of available ITNs in country *i* throughout 2020, and $V_i(t)$ represents the mean value of 16-year VCAP in time interval *t* of each year.

As the number of newly reported COVID-19 cases $\Delta _{R_i}(t)=R_i(t)-R_i(t-1)$ increases, it is assumed that the distribution of ITNs will be disrupted accordingly. Moreover, when the COVID-19 becomes serious (e.g., the number reaches a threshold value $\tau$), the distribution of ITNs will be suspended. Mathematically, we assume that the number of distributed ITNs in time interval *t*, $D_i(t)$, is inversely proportional to the number of reported COVID-19 cases. Thus, we have:

4 $$\begin{aligned} D_i(t) = {\left\{ \begin{array}{ll} (1-\Delta _{R_i}(t)/\tau )\Delta _i(t), &{} \text {if } \Delta _{R_i}(t) < \tau , \\ 0, &{} \text {otherwise}. \end{array}\right. } \end{aligned}$$

Let $D_i(1:t)= \sum _t D_i(t)$ represent the cumulative number of distributed ITNs from the first time interval to the *t*th interval in 2020. Then, the newly increased ITN coverage rate till time interval *t* becomes $D_i(1:t)/N_i$, where $N_i$ is the population size of country *i*. For case studies in each of the four African countries, the threshold value $\tau$ is set to be the number of reported cases when various NPIs are implemented.

The disruption or cessation of distribution of ITNs may reduce the expected ITN coverage in a country, which may lead to the increase in human feeding rate *a*, as well as the transmission potential of malaria. Denote $C_i$ as the ITN coverage rate in country *i* before 2020. In this study, we treat $C_i$ as a reference value, which corresponds to human feeding rate of the baseline value of VCAP. Then, if all available ITNs are distributed as expected, the relative change of human feeding rate can be estimated as follows:

5 $$\begin{aligned} r_i^{exp}(t) = \frac{1-\alpha (C_i+\Delta _i(1:t)/N_i)}{1-\alpha C_i}, \end{aligned}$$

where $\alpha$ indicates the efficiency of ITNs against mosquito bites. According to the definition of vectorial capacity, the expected transmission potential of malaria at time interval *t* can be calculated as:

6 $$\begin{aligned} V_i^{exp}(t) = (r_i^{exp}(t))^2\cdot V_i(t). \end{aligned}$$

Similarly, if the distribution of ITNs is disrupted, the relative change of human feeding rate is:

7 $$\begin{aligned} r_i^{dis}(t) = \frac{1-\alpha (C_i+D_i(1:t)/N_i)}{1-\alpha C_i}, \end{aligned}$$

and the transmission potential becomes:

8 $$\begin{aligned} V_i^{dis}(t) = (r_i^{dis}(t))^2\cdot V_i(t). \end{aligned}$$

In this study, we set $\alpha =1$. Note that if the ITN distribution is disrupted, we have $V_i^{exp}(t)<V_i^{dis}(t)<V_i(t)$. In doing so, we can quantify the relative change of malaria transmission potential due to the negative effects of pandemic COVID-19 on the ITN campaigns in each country.

## Results

### Reconstruction of COVID-19 dynamics in each country

**Fig. 2:**
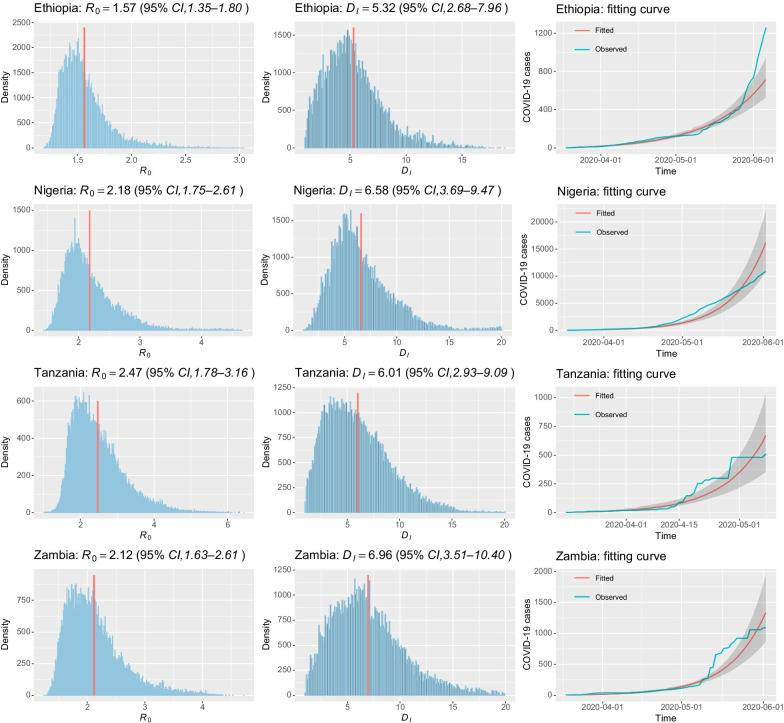
The inferred epidemiological parameters and the fitting results in Ethiopia, Nigeria, Tanzania, and Zambia. The parameters $R_0$ and $D_I$ are estimated using the particle Markov Chain Monte Carlo method by fitting the time series of cumulative number of reported COVID-19 cases in each country till June 2, 2020. The first two columns shows the density and mean values of $R_0$ and $D_I$ in each country. The third column shows the fitting curves (red lines) and the cumulative cases (blue lines) of COVID-19 in each country

In this study, we adopt the PMCMC method to estimate the epidemiological parameters, i.e., the basic reproduction number $R_0$ and the average duration of infection $D_I$, by fitting the cumulative number of reported COVID-19 cases in each country till June 2, 2020. Figure 2 demonstrates the inferred model parameters and the fitting results in Ethiopia, Nigeria, Tanzania, and Zambia, respectively. The sampled posterior distributions of $R_0$ and $D_I$ are shown in the first two columns. It can be observed that in different countries, epidemiological characteristics varies greatly. In Ethiopia, the estimated basic reproduction number $R_0$ is 1.57 [95% confidence interval (*CI*) 1.35–1.8] and the average duration of infection $D_I$ is 5.32 days (95% *CI* 2.68–7.96); In Nigeria, the estimated $R_0$ is 2.18 (95% *CI* 1.75–2.61) and the estimated $D_I$ is 6.58 days (95% *CI* 3.69–9.47); In Tanzania, the estimated $R_0$ is 2.47 (95% *CI* 1.78–3.16) and $D_I$ is 6.01 days (95% *CI* 2.93–9.09); In Zambia, the estimated $R_0$ is 2.12 (95% *CI* 1.63–2.61) and $D_I$ is 6.96 days (95% *CI* 3.51–10.40). The bell-shaped posterior distribution of parameter samples indicates a good estimation of model parameters in the four countries. In reality, at the beginning of the epidemic, the COVID-19 cases are not reported on a daily basis. Therefore, there are many zeros in the time series of newly reported cases. It is noteworthy that zero cases in a day do not mean that there are no new infections. More likely, new infections in that day will be reported a few days later. In this case, it becomes difficult to accurately fit the true number of reported cases. The third column in Fig. 2 shows the fitting curves with 95% confidence interval of the cumulative number of COVID-19 cases in each country. We measure the goodness of fit using the root mean squared error (RMSE), the value of which is 111.22 for Ethiopia, 1263.36 for Nigeria, 66.69 for Tanzania, and 124.37 for Zambia. Because the COVID-19 infections in these countries are not reported in time, the values of RMSE are relatively high. However, it can be observed that the fitting belts generated by the PMCMC algorithm can well cover the time series of cumulative cases in most situations. Moreover, it must be pointed out that no new COVID-19 cases have been reported in Tanzania since May 8, 2020. Although the fitting curve can well cover the cumulative number of reported cases, the basic reproduction number $R_0$ of Tanzania is likely underestimated. While in Ethiopia, the number of reported cases dramatically increases after May 29, 2020. As shown in Fig.  2, the fitting curve is slightly lower than the cumulative number of reported COVID-19 cases. As a consequence, $R_0$ of Ethiopia may also be underestimated.

### Simulation of COVID-19 epidemic under different non-pharmaceutical interventions

**Fig. 3:**
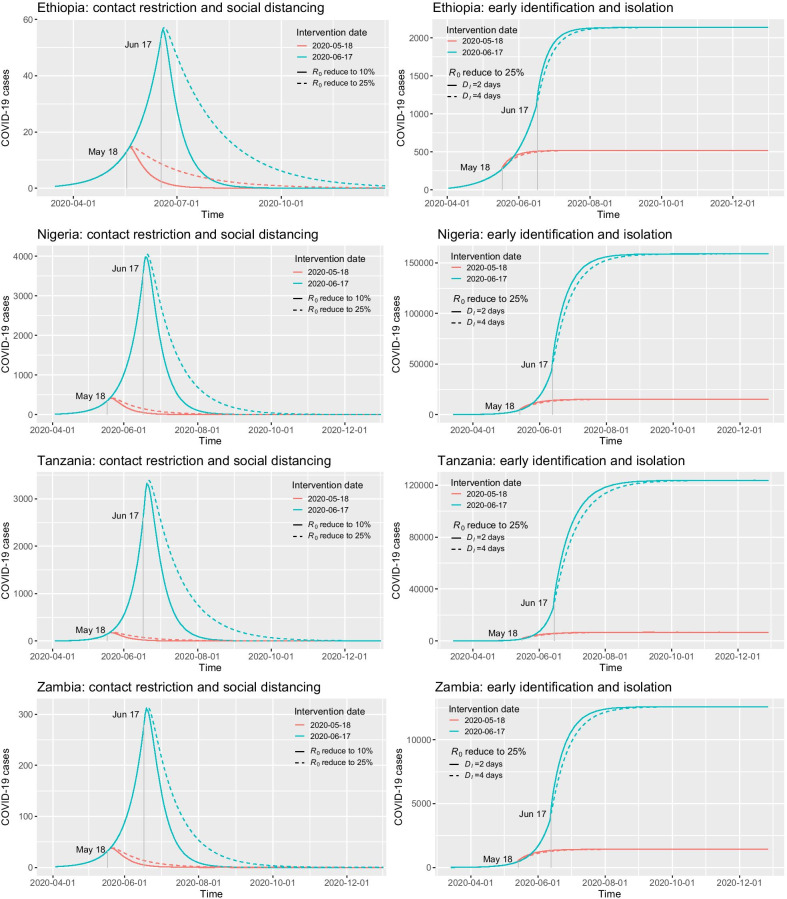
The simulation of COVID-19 epidemic in Ethiopia, Nigeria, Tanzania, and Zambia under different NPIs implemented on May 18 and June 17, 2020. The left column shows the number of newly-reported COVID-19 cases over time under the NPI of contact restriction and social distancing, where $R_0$ is reduced to 25% and 10% of its original value. The right column shows the cumulative number of reported COVID-19 cases under combined NPIs, where $R_0$ is reduced to 25% and $D_I$ is reduced to two or four days

We simulate the epidemic curves of COVID-19 in Ethiopia, Nigeria, Tanzania, and Zambia under various types of NPIs implemented on May 18 and June 17, 2020. First, the NPI of contact restriction and social distancing is evaluated by reducing the basic reproduction number $R_0$ to 25% and 10% of its estimated value, while keeping the duration of infection $D_I$ unchanged. In doing so, the infectious rate $\beta$ is reduced to 25% and 10%, respectively. It is noteworthy that both NPIs are very stringent. For example, even if $R_0$ is reduced to 25%, it becomes $R_0=0.6175$ in Tanzania and $R_0 = 0.3925$ in Ethiopia. Intuitively, the more stringent the intervention measures, the easier it is to control the epidemic, and its influence on malaria intervention measures will gradually be weakened. As shown in the left column of Fig. 3, the solid lines always reach zero cases earlier than the dashed lines. Moreover, the earlier the intervention is implemented, the smaller the peak number of infections, and the sooner the peak comes. Figure 3 indicates that if the NPIs are implemented one month late, the number of infections will increase exponentially.

The second column of Fig. 3 shows the cumulative number of reported COVID-19 cases under combined NPIs, where $R_0$ is reduced to 25%, and $D_I$ is reduced to two or four days. Comparing with the NPI of reducing $R_0$ to 25% only, the combined NPIs cannot decrease the total number of infections. Nevertheless, when the duration of infection $D_I$ is smaller (e.g., $D_I=2$ days), the epidemic will be controlled faster than the NPI with a larger $D_I$. Meanwhile, the peak of infection (i.e., the inflection point) will also come earlier. It can be observed from Fig. 3 that the combined NPI with $D_I=2$ (i.e., the solid lines) can control the epidemic faster than that with $D_I=4$ (i.e., the dashed lines) when they were implemented on the same day. In this case, if the ITNs are distributed throughout the year, the time duration it will be affected by the epidemic will be shorter. Hence, the combined NPIs may reduce the impact of COVID-19 epidemic on the distribution of ITNs, and further the transmission potential of malaria.

### Impact of COVID-19 pandemic on malaria transmission potential

**Fig. 4:**
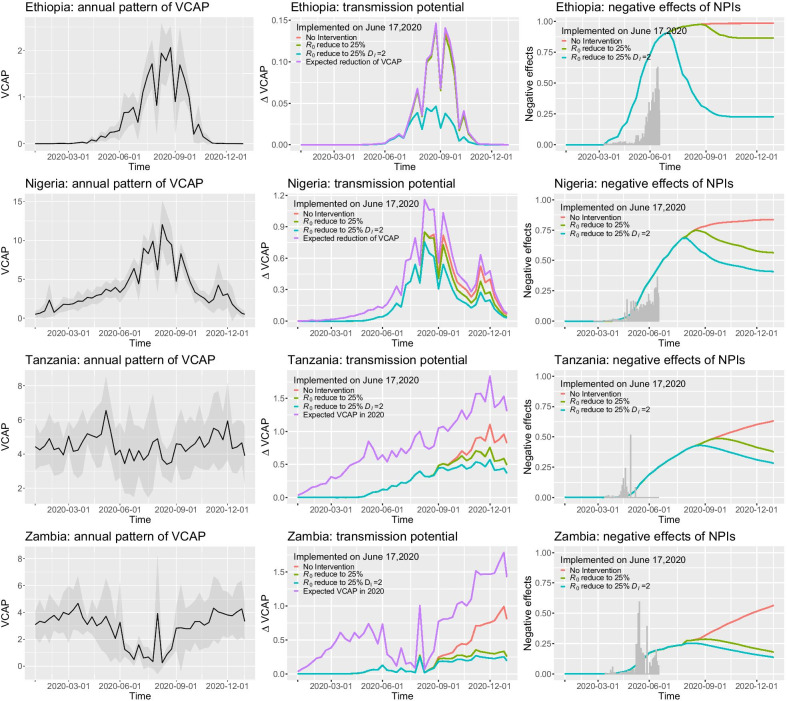
The impact of various NPIs for COVID-19 pandemic on the transmission potential of malaria in 2020. The first column shows the annual pattern of VCAP and corresponding confidence interval in each country, which is treated as malaria transmission potential through a year. The second column shows the reduction of transmission potential through ITN distribution under various NPIs implemented on June 17, 2020. The third column shows the negative effects of COVID-19 pandemic under various NPIs that cannot achieve the desired reduction in transmission potential of malaria. The gray bars are the real number of reported COVID-19 cases

In this study, we adopt the notion of vectorial capacity to evaluate the transmission potential of malaria. To quantify the impact of COVID-19, we first analyze the annual pattern of the transmission potential of malaria. The first column in Fig. 4 shows the mean values of VCAP and corresponding confidence intervals in each country. It can be observed that the annual patterns of VCAP in different countries vary greatly due to different meteorological conditions throughout a year. Intuitively, if the COVID-19 epidemic occurs during the malaria transmission season, it will seriously disrupt health services for routine malaria intervention and control. Therefore, we further quantify the expected reduction of malaria transmission potential without COVID-19 pandemic [i.e., $V_i(t)-V_i^{exp}(t)$] based on ITNs available in each country in 2020. Specifically, based on the simulated COVID-19 epidemic under various NPIs, we estimate the reduction of malaria transmission potential throughout 2020. The results are shown in the second column in Fig. 4. The purple lines show the expected reduction of malaria transmission potential when the COVID-19 pandemic did not occur. It can be observed that in Ethiopia and Nigeria, the trend of reduction is generally consistent with the annual pattern of VCAP. The reason is that based on our assumption, most ITNs in Ethiopia and Nigeria will be distributed during the malaria transmission reason from June to October. While in Tanzania and Zambia, the reduction of malaria transmission potential will gradually increase in the second half of the year. This is because some ITNs will be distributed in the first half of the year, and the cumulative number of ITNs will play an important role in reducing the malaria transmission potential in the second half of the year.

In addition, the second column in Fig. 4 also shows the difference of VCAP compared to the expected VCAP (i.e., $\Delta V_i(t) = V_i^{dis}(t)-V_i^{exp}(t)$) caused by various NPIs implemented on June 17, 2020. It can be observed that in the first quarter of 2020, there is no difference between the red, yellowgreen, and turquoise lines in each country because no COVID-19 cases are reported. As the COVID-19 epidemic becomes more and more serious, the difference $\Delta V_i(t)$ begins to grow in Tanzania and Zambia due to its negative effect on the distribution of ITNs. On the contrary, in Ethiopia and Nigeria, the difference becomes large during the malaria season (i.e., from June to October) due to the simultaneous transmission of COVID-19 and malaria. When the COVID-19 epidemic is controlled and ITNs are distributed as scheduled after October, the malaria transmission season is over. As a consequence, the distributed ITNs cannot contribute to malaria intervention and control this year anymore.

The third column in Fig. 4 shows the negative effects of COVID-19 pandemic under various NPIs that cannot achieve the desired reduction in the transmission potential of malaria. Here, the negative effect is defined as $(V_i^{dis}(t)-V_i^{exp}(t))/(V_i(t)-V_i^{exp}(t))$, which indicates the proportion of VCAP reduction that cannot completed as expected. In the first half of 2020, as the number of reported cases increases in each country (i.e., the gray bars), the negative effect begins to increase accordingly. However, because no NPIs are implemented before June 17, 2020, the negative effects of different NPIs are all the same. Therefore, the three lines are overlapped. After June 17, 2020, different NPIs are implemented. It can be observed that the more stringent the NPI, the more it can achieve the desired goal. When no NPIs are implemented, the negative effect is the largest (see the red curves in Fig. 4). When $R_0$ is reduced to 25% on June 17, 2020, the negative effect is alleviated after September. The reason is that the COVID-19 epidemic cannot be controlled before September. However, when the combined NPI (i.e., $R_0$ is reduced to 25% and $D_I=2$) is implemented, the epidemic can be controlled much earlier. Hence, the negative effect is alleviated before September, and the negative effects is the smallest (see turquoise lines in Fig. 4).

## Discussion

As the COVID-19 pandemic spreads rapidly in Africa, there is an urgent need to mitigate the negative impact of the coronavirus in malaria-endemic countries and contribute towards a syndemic of COVID-19 and malaria intervention. To achieve this goal, the first priority is to assess the epidemiological characteristics of the COVID-19 pandemic in each country. Because populations in different countries have different contact patterns, the epidemiological parameters (e.g., the basic reproduction number $R_0$) of COVID-19 may also vary in different countries [24, 25]. Even in the same country, the epidemiological parameters measured with different data at different stages may also be different. For example, Li et al. have analyzed the first 425 diagnosed COVID-19 cases in Wuhan and found that $R_0$ is about 2.2 [1]. Then, Wu et al. have combined international aviation data and domestic Tencent mobile data to estimate that the $R_0$ of COVID-19 is around 2.68 [26]. Tian et al. have analyzed through different stages of the COVID-19 epidemic in China: before the emergency response was initiated on January 23, $R_0$ was 3.15; since January 23, due to the expansion of the scope of prevention and control measures, the $R_0$ in different provinces has declined; when the intervention coverage reaches 95%, the average $R_0$ drops to 0.04 [27]. In this study, based on the time series of COVID-19 cases as of June 2, 2020, we have employed the classical SEIR model to simulate the transmission dynamics of COVID-19, and adopted a PMCMC method to estimate the epidemiological parameters by fitting the cumulative number of reported COVID-19 cases in each country. Comparing with the early stage of COVID-19 spread in China, the estimated $R_0$ and $D_I$ are both in a reasonable range [1]. However, the epidemiological characteristics of the four countries are different. Therefore, to assess the syndemic of COVID-19 and malaria intervention, it would be necessary to simulate the various COVID-19 responses in each country separately.

To simulate the impact of COVID-19 response, in this study, we have conduct scenarios analysis on two groups of NPIs: (1) contact restriction and social distancing by reducing $R_0$ to 25% or 10% of its original value while keeping $D_I$ unchanged, and (2) early identification and isolation of new cases by reducing $D_I$ to two or four days. The results have shown that the former NPIs can effectively reduce the scale of the epidemic, while the latter can shorten the duration of the epidemic. In this case, a combination of them should be a better response to the COVID-19 epidemic. In addition to the type of NPIs, when to intervene is much more important. Our simulation results have also shown that if the NPIs are implemented one month earlier (see Fig. 3), the scale of infection will be greatly reduced, and the epidemic will be effectively controlled much earlier. Because of this, the early adoption of comprehensive NPIs like China is essential to timely and effective control of COVID-19 epidemic.

Different from the COVID-19, malaria is a mosquito-borne infectious disease, whose transmission depends on various impact factors, such as climate [28, 29], human movement [30–33], and socio-economic factors [34, 35]. In this study, we adopt the notion of vectorial capacity to represent the transmission potential of a mosquito population in the absence of *Plasmodium*. Existing studies have shown that the value of VCAP can be estimated based on meteorological factors, such as precipitation and temperature [19]. Because different countries have different meteorological conditions, the patterns of VCAP vary greatly through a year (see Fig. 4). In Ethiopia and Nigeria, the high-risk season for malaria transmission is from June to October. In contrast, Zambia has a relatively low risk of transmission during this period. While in Tanzania, the risk of transmission is relatively stable throughout a year.

To assess the syndemic of COVID-19 and malaria intervention, it would be necessary to consider whether they spread together over a period of time. On the one hand, if the COVID-19 occurred during the malaria transmission season, it would have a great impact on the allocation of resources for malaria prevention and control, thereby greatly reducing the expected effect of malaria intervention. For example, in Ethiopia and Nigeria, even with strict prevention and control measures, it is still impossible to contain the COVID-19 epidemic before the malaria transmission season (from June to October). In this case, the negative impact of the COVID-19 epidemic will be amplified due to the cumulative effect on unallocated ITNs (see the third column in Fig. 4). To jointly address endemic malaria and pandemic COVID-19 in such countries, as suggested by World Health Organization, it would be necessary to maintain core malaria control services (e.g., ITN distribution) while implementing stringent NPIs for COVID-19. On the other hand, if the simulated peak of the COVID-19 epidemic is not in the malaria transmission season (e.g., Zambia), it will have little impact on the expected effects of malaria intervention in a short period. Even so, the cumulative effects still can be amplified in the future. Therefore, to avoid the resonance of COVID-19 and malaria, the best way is to do everything possible to contain COVID-19 before the next malaria transmission season. Otherwise, it would be necessary to allocate as many resources as possible for malaria prevention and control before the arrival of the next malaria transmission season. In summary, in African countries where malaria is considered to be epidemic, strategically maintaining core malaria control services such as the distribution of ITNs, is crucial important during the COVID-19 pandemic.

Limited by the data available at the moment, the proposed method in this study still has several limitations that are worthy of being improved in the future. First, without identifying asymptomatic infections, it would be difficult to quantitatively assess the impact of asymptomatic infections on the COVID-19 epidemic in the real world. Second, asymptomatic infections can also increase the difficulty of case identification and result in the underreporting of COVID-19 cases. This will lead to an underestimation of the severity of the COVID-19 epidemic. Nevertheless, in reality, the decisions to reduce or suspend the distribution of ITNs are based on the reported COVID-19 cases. Therefore, even the severity of the COVID-19 epidemic is underestimated, it may have little impact on assessing the impact of NPI response on the potential risk of malaria transmission as long as the number of reported cases can be correctly estimated. In the future, when information about asymptomatic infections is available, it would be possible to tackle the underreporting issue by constructing more precise transmission models.

## Conclusions

In this study, we have presented a data-driven method to assess the syndemic of COVID-19 and malaria intervention in four malaria-endemic countries in Africa: Ethiopia, Nigeria, Tanzania, and Zambia. To achieve this goal, we have first estimated the epidemiological parameters, i.e., the basic reproduction number $R_0$ and the duration of infection $D_I$, based on the time series of reported COVID-19 cases of each country. Then, we have simulated COVID-19 epidemic under two groups of NPIs: (1) contact restriction and social distancing, and (2) early identification and isolation of cases. Based on the simulated epidemic curves of COVID-19, we have quantified the impact of COVID-19 response on the distribution of ITNs in each country. Finally, we have further assessed the negative effects of COVID-19 pandemic and various NPIs on the transmission potential of malaria using the notion of vectorial capacity. The results and findings in this paper provide a way to jointly address COVID-19 and malaria transmission, as well as to efficiently utilize limited health services in malaria-endemic countries in Africa.

## Data Availability

Please contact author for data requests.

## Abbreviations

ITN: Insecticide-treated net
PMCMC: Particle Markov chain Monte Carlo
NPI: Non-pharmaceutical intervention
VCAP: Vectorial capacity
SEIR: Susceptible–exposed–infectious–removed
MIS: Malaria indicator survey
PMI: President’s malaria initiative

## Footnotes

1 A WHO Statement: https://www.who.int/emergencies/diseases/novel-coronavirus-2019/question-and-answers-hub/q-a-detail/malaria-and-the-covid-19-pandemic.
